# Feasibility of Measuring Smartphone Accelerometry Data During a Weekly Instrumented Timed Up-and-Go Test After Emergency Department Discharge: Prospective Observational Cohort Study

**DOI:** 10.2196/57601

**Published:** 2024-09-04

**Authors:** Brian Suffoletto, David Kim, Caitlin Toth, Waverly Mayer, Sean Glaister, Chris Cinkowski, Nick Ashenburg, Michelle Lin, Michael Losak

**Affiliations:** 1 Department of Emergency Medicine, Stanford University, 300 Porter Drive, Palo Alto, CA, 94020, United States, 1 650-723-6576, 1 650-723-0121

**Keywords:** older adult, older adults, elder, elderly, older person, older people, ageing, aging, gait, balance, fall, falls, functional decline, fall risk, fall risks, mobility, phone, sensors, patient monitoring, monitoring, emergency department, emergency departments, ED, emergency room, ER, discharge, mobile application, mobile applications, app, apps, application, applications, digital health, digital technology, digital intervention, digital interventions, smartphone, smartphones, prediction, mobile phone

## Abstract

**Background:**

Older adults discharged from the emergency department (ED) face elevated risk of falls and functional decline. Smartphones might enable remote monitoring of mobility after ED discharge, yet their application in this context remains underexplored.

**Objective:**

This study aimed to assess the feasibility of having older adults provide weekly accelerometer data from an instrumented Timed Up-and-Go (TUG) test over an 11-week period after ED discharge.

**Methods:**

This single-center, prospective, observational, cohort study recruited patients aged 60 years and older from an academic ED. Participants downloaded the GaitMate app to their iPhones that recorded accelerometer data during 11 weekly at-home TUG tests. We measured adherence to TUG test completion, quality of transmitted accelerometer data, and participants’ perceptions of the app’s usability and safety.

**Results:**

Of the 617 approached patients, 149 (24.1%) consented to participate, and of these 149 participants, 9 (6%) dropped out. Overall, participants completed 55.6% (912/1639) of TUG tests. Data quality was optimal in 31.1% (508/1639) of TUG tests. At 3-month follow-up, 83.2% (99/119) of respondents found the app easy to use, and 95% (114/120) felt safe performing the tasks at home. Barriers to adherence included the need for assistance, technical issues with the app, and forgetfulness.

**Conclusions:**

The study demonstrates moderate adherence yet high usability and safety for the use of smartphone TUG tests to monitor mobility among older adults after ED discharge. Incomplete TUG test data were common, reflecting challenges in the collection of high-quality longitudinal mobility data in older adults. Identified barriers highlight the need for improvements in user engagement and technology design.

## Introduction

Each year, millions of older adults are discharged from emergency departments (EDs) across the United States [1]. A growing body of evidence indicates that these individuals face high risks of adverse outcomes after ED discharge, including falls [2] and functional decline [3]. While guidelines aim to identify those at risk of poor outcomes [4], existing fall risk screening tools using data at the time of the ED encounter have limited ability to predict which patients will fall [2].

One way to improve the identification of older adults at risk for falls is to incorporate remote patient monitoring (RPM) of mobility into postdischarge care. Mobility, which includes gait and balance functions, requires the integration of sensory input, motor planning, and coordination. Gait alterations and balance issues are common in individuals aged 65 years and older [5,6] and both significantly increase the risk of falls [7]. RPM of gait and balance in home settings may identify mobility problems that are not readily apparent in controlled settings [8]. Additionally, RPM allows for examination of within-person changes over time, which can improve the discrimination of predictive models [9]. However, the success of any RPM depends heavily on the practicality and usability of the technology for older adults.

Numerous tools exist that allow for RPM of gait and balance, including external sensors (eg, cameras and force plates) and wearable sensors (eg, smartphones). Unlike external sensors, which require potentially expensive hardware and installation, wearable sensors are portable; cheaper; and in the case of smartphones, near ubiquitous [10]. Smartphones are equipped with inertial measurement units, typically composed of an accelerometer and a gyroscope. These sensors enable smartphones to accurately monitor gait mechanics [11], which can identify individuals at higher risk of falls [12]. Despite these capabilities, the potential of smartphone-based RPM of mobility after ED discharge remains largely unexplored. Describing and understanding the drivers of participants’ engagement with RPM in research is necessary to determine the success of future real-world implementation of RPM in clinical services.

This study aimed to assess the feasibility of having older adults provide weekly accelerometer data from an instrumented Up-and-Go test [12] over an 11-week period after ED discharge. The Up-and-Go test (commonly referred to as the Timed Up-and-Go [TUG] test) involves an individual getting up from a chair, walking forward, turning, returning to the chair, and sitting. The TUG test was chosen because it is simple and quick and evaluates several key risk factors, including gait and balance, in a single assessment. Instrumented TUG tests using body-worn sensors can identify distinct gait patterns and balance issues [13], are validated against standard kinematic measures [14], and can distinguish between individuals who have experienced falls and those who have not [12]. To our knowledge, no prior study has reported on adherence to at-home TUG tests.

The primary focus of this study was on adherence, defined as the degree to which the user followed the program as designed [15], which involved completing weekly at-home instrumented TUG tests. Secondary aims focused on data quality, app usability, safety during at-home functional tasks, and barriers to adherence. Data quality is essential for generating meaningful gait and balance features, and various user-specific factors can negatively impact it [16,17]. Findings from this study provide foundational information for developing age-friendly RPM technologies, anticipating the increasing demand for improved postdischarge transitional care among older adults in the coming years.

## Methods

### Study Design

This study was a single-center, prospective, observational, cohort study of ED patients.

### Ethical Considerations

This study was approved by the Stanford University institutional review board (IRB #64194), and written informed consent was obtained from all participants. The data collection app (GaitMate) was built using Stanford’s Cardinal Kit and all data were stored in a secure Firebase account managed by Stanford Research IT. Participants were required to log in using a unique ID with each app task; this ID was the sole identifier linking them to the data. Compensation of up to US $90 was offered to study participants.

### Study Setting and Participants

A convenience sample of patients was recruited by research associates (RAs) from a single academic ED with an annual volume of 100,000 visits. Patients were eligible for participation in the study if they were 60 years of age or older, were to be discharged home, and owned an iPhone. We excluded from the study patients currently living in a nursing home, patients with limited English proficiency, patients who could not walk unaided (ie, walking without an assistive aid), and patients without the capacity to provide informed consent. If a participant moved to a nursing home after the time of consent but during the follow-up period, they remained in the study. The study is reported in accordance with the STROBE (Strengthening the Reporting of Observational Studies in Epidemiology) guidelines for reporting observational studies [18].

### Study Procedures

#### Overview

In the ED, RAs helped participants download the GaitMate app and led them through the self-report and functional baseline assessments. For 11 weeks after ED discharge, patients were asked to complete a weekly TUG test and to report any falls through the app. At 12 weeks after discharge or enrollment, we attempted to reach all participants by phone to collect data on perceived ease of app use and safety during at-home TUG tests.

#### Onboarding and Baseline Assessments

In the ED, each participant was guided by an RA to download the GaitMate from the Apple App Store and was assigned a unique ID they used to access the app thereafter. Next, participants were led by the RA through baseline survey questions. Subsequently, participants were presented with an in-app instructional video detailing the TUG test [19]. The RA then demonstrated the TUG procedures and helped participants complete the first task in the ED, which involved the participant standing up from a seated position, walking 2 meters, turning around, returning to the chair, and sitting down. To augment the assessment, we provided participants a waist belt equipped with a pouch to securely hold the smartphone during the TUG test. This setup enabled us to collect 3-axis accelerometer data from the phone, positioned near the body’s center of mass, thereby allowing us to estimate spatial characteristics of steps. Participants who had difficulty placing the phone in the pouch and rotating to their back were instructed to keep it in the front. Multimedia Appendix 1 provides an example of the waist belt and phone placement. We chose a 2-meter walking distance for the TUG test instead of the original 3 meters given that there is limited space in the ED to perform the TUG test and concerns about the unobstructed space in patients’ homes. After completing the task, participants removed the phone from the pouch and pressed the “DONE” button, prompting the transmission of the accelerometer data directly to institutional research servers for analysis. Multimedia Appendix 2 presents screenshots of the GaitMate app.

#### Home-Based Assessments

Each Sunday at 12 PM for 11 weeks following ED discharge, participants received a GaitMate notification prompting them to complete their weekly TUG test. After entering their ID, they were instructed to tap the “Weekly Check-in” button. Participants then viewed an instructional video on the task and were asked to complete a safety checklist. This included verification of having cleared a walking space, having set up a chair to one side, having put on regular footwear and the belt pouch, and having someone present to assist if needed. After completing the checklist, participants were asked to tap the “READY” button to start recording data. In addition, participants could log any fall by tapping the “Report a fall” button on the main screen, which would prompt the participant to record (1) the date of a fall, (2) time of fall, and (3) injury associated with fall. At-home TUG test completion was monitored by RAs. When a participant missed 3 weeks in a row, RAs attempted to reach that participant by email once and then phone to probe barriers to completing at-home tasks with open-ended questions. If a participant lost their ID, they were provided with the contact information of study investigators.

#### Follow-Up Phone Call

At 11 weeks following ED enrollment, a trained RA called all participants by phone, making up to 3 attempts before marking the participant as lost to follow-up. Follow-up phone calls assessed falls over the study period and whether any occurred during at-home TUG tests, perceived ease of app use, and safety while completing at-home TUG tests.

### Measures

#### Baseline Assessments

To understand how ED patients who enrolled differ from those who did not, we collected limited information (ie, age, sex, chief complaint, and illness severity) on all prescreened patients. To understand the baseline characteristics of our participants, we additionally recorded race, ethnicity, ED chief complaints, and active medical problems.

#### Functional Task Completion

**Figure 1.**
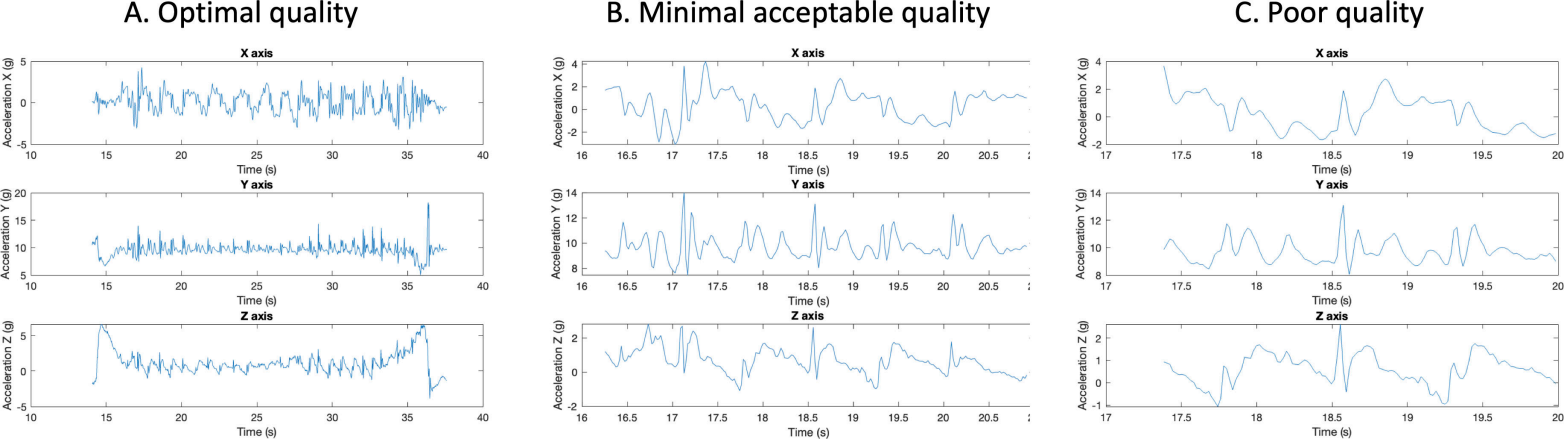
Examples of accelerometer data quality. (A) Optimal quality: there are clear sit-to-stand, walk-away, walk-back, and stand-to-sit portions of the gait task as well as displacements indicative of steps. (B) Minimal acceptable quality: there is a truncated segment with a grouping of at least 3 steps. (C) Poor quality: there is a severely truncated segment without 3 consecutive displacements indicative of steps.

The primary outcome was the completion of the weekly TUG tests, defined as any transmitted accelerometer data for a given week. The secondary outcome was accelerometer data quality, assessed by 2 RAs independently, with the lead author serving as an arbiter when there was disagreement. All data-quality assessors have extensive training and experience segmenting and generating gait and balance features using accelerometer data. Each task submission was classified as one of four categories: (1) *Optimal data quality*, defined as unambiguous visual segmentation of the data into the sit-to-stand, walk-away, walk-back, and stand-to-sit portions of the gait task; (2) *Minimal acceptable data quality*, defined as whether at least 3 steps during the walk-away or walk-back segment could be visually identified; (3) *Poor data quality*, defined as cases that did not meet category 1 or 2 but where some data were transmitted; and (4) *Missing*, if no data were transmitted for active participants. Figure 1 illustrates accelerometer data in the first 3 categories.

#### Follow-Up Assessments

To assess falls during the study period, we asked “In the past 3-months, how many times have you fallen?” followed by “Did any of these falls result in injury?” and “Did any of these falls result in the need to seek acute medical care?” Finally, we asked “Can you recall the situation that led to the fall?” To understand how participants perceived the GaitMate app, we asked “How much do you agree or disagree with the following statements: (1) Overall, the app was easy to use. (2) I felt safe completing the gait task at home.” Response options ranged on a 5-point scale from strongly agree to strongly disagree. For simplicity, we collapsed the 5-point scale into 3 categories: agree, disagree, and neither agree nor disagree.

### Data Analyses

For our primary analysis, we first calculated the TUG test submission rates by week and by participant. We then categorized participants into low adherence (0%-49%), moderate adherence (50%-99%), or perfect adherence (100%) and used ordered logistic regression models to examine whether the adherence was associated with participant characteristics of age, sex, race, active medical problems, and chief complaint category. In secondary analyses, we calculated the distribution of data-quality categories for each week and by participant. To quantify older adult perceptions of GaitMate usability and safety, we calculated the percentage who agreed with the usability and safety statements. To understand barriers to adherence, we described qualitative feedback from participants when they reported difficulty with the app.

## Results

### Study Enrollment and Retention

**Figure 2.**
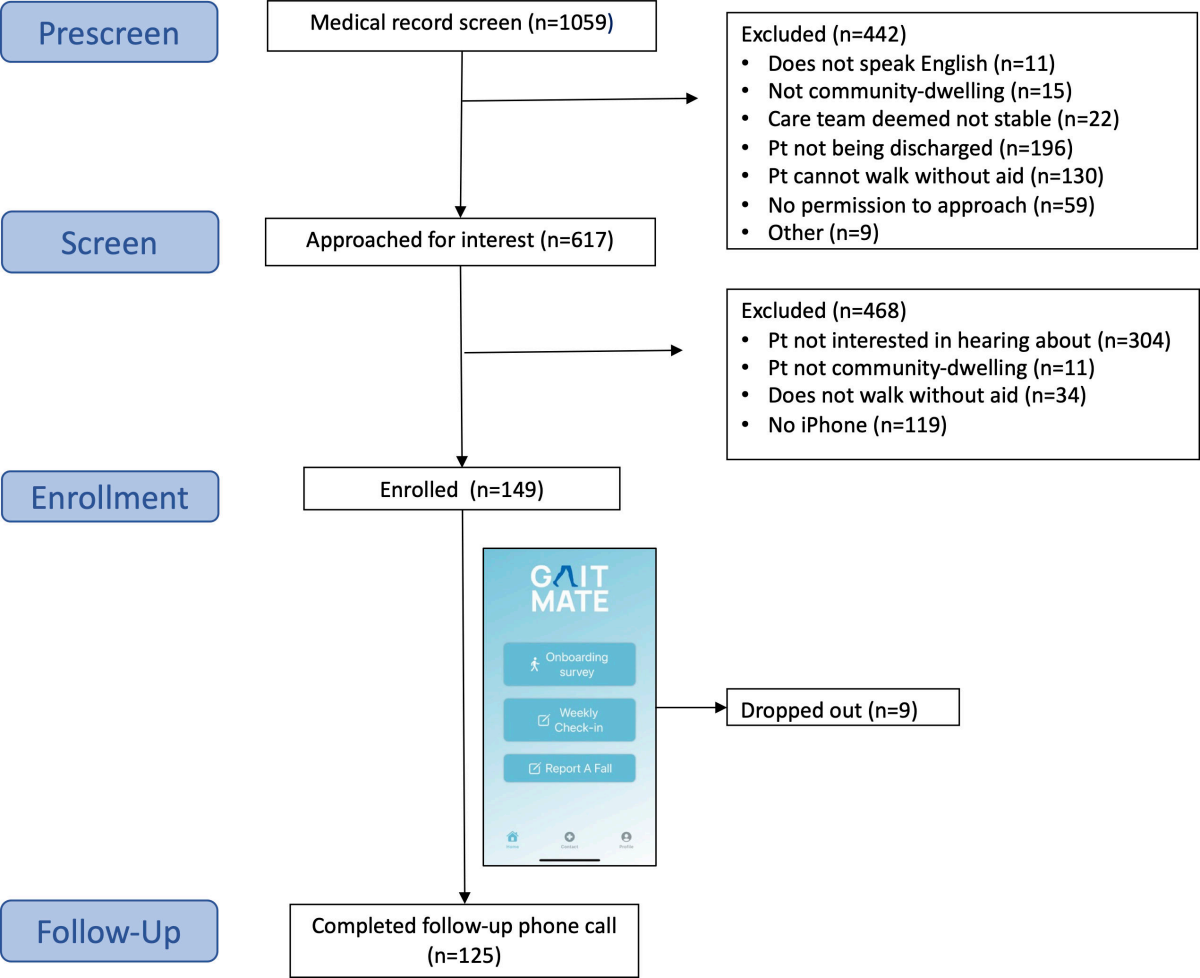
Participant screening, enrollment, and follow-up. Pt: patient.

Figure 2 outlines the flow of patients from enrollment through follow-up. From December 5, 2022, to August 9, 2023, we identified 1059 ED patients from the medical record who were aged 60 years or older. We excluded 442 ED patients after discussion with ED providers, with the majority excluded because they either were not being discharged to home (196/442, 44.3%) or could not ambulate unaided (130/442, 29.4%). We approached 617 patients for screening, among whom 468 (75.9%) were not interested in study participation, resulting in the recruitment of 149 participants. Common qualitative reasons for nonparticipation included feeling too sick, too busy, or lack of interest. There were no statistical differences in patient age, sex, or Emergency Severity Index between those who agreed to participate and those who did not. Of the 149 participants, 9 (6%) dropped out of the study: 4 (2.7%) participants prior to completing baseline assessments, 4 (2.7%) more participants in week 1, and 1 (0.7%) participant in week 7. Follow-up assessments at week 12 were completed by 125 (89.3%) of 140 retained participants.

### Participant Characteristics

Table 1 demonstrates the descriptive characteristics of the 149 enrolled participants. The mean age of the enrolled participants was 72.3 (SD 8.2) years, and the majority (n=91, 61.1%) were male. Almost half of participants (n=69, 46.3%) had fallen in the past year, indicating a fall-vulnerable cohort. The presenting ED complaints were highly varied, with only 10% (n=15) presenting for fall-related care. Participants had the comorbidity profile expected of older adults, with 63.8% (n=95) having high blood pressure, 40.9% (n=61) having heart disease, and 51.7% (n=77) reporting a past orthopedic surgery.

**Table 1.** Baseline characteristics.

Variable	Enrolled participants (n=149)
**Demographics**	
Age (year), mean (SD)	72.3 (8.2)
Female, n (%)	58 (38.9)
White, non-Hispanic, n (%)	98 (65.8)
**Fall history, n (%)**	
Any fall in the past year	69 (46.3)
**ED^a^ chief complaint category, n (%)**	
Cardiac	27 (18.1)
Respiratory	5 (3.4)
Gastrointestinal	20 (13.4)
Neurological	31 (20.8)
Genitourinary	6 (4)
Fall	15 (10.1)
Musculoskeletal	22 (14.8)
Other	23 (15.4)
**Medical history, n (%)**	
Cardiac or heart disease	61 (40.9)
Respiratory problems	26 (17.4)
Gastrointestinal problems	47 (31.5)
Vision conditions	29 (19.5)
Endocrine conditions (eg, diabetes)	51 (34.2)
Motion sickness or vertigo	54 (36.2)
High blood pressure	95 (63.8)
Orthopedic surgeries	77 (51.7)

a ED: emergency department.

### Task Completion Rates

**Figure 3.**
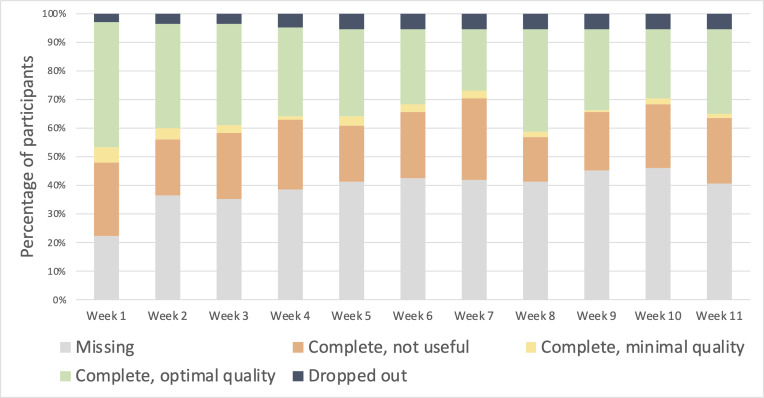
Gait data completeness and quality over the study period.

Figure 3 summarizes task completion and data quality over the course of the study. Accelerometer data from 55.6% (912/1639) of weekly TUG tests was transmitted over 11 weeks after discharge. The completion rates declined from 59.7% (89/149) in week 1 to 53.7% (80/149) in week 11. Overall, 23.4% (35/149) of participants completed TUG tests in all 11 weeks, 18.8% (28/149) did not complete any TUG tests, and 57.7% (86/149) of participants completed TUG test in at least 6 weeks. Adherence was similar across sex, race and ethnicity, active medical problems, and chief complaint categories.

### Data Quality

**Figure 4.**
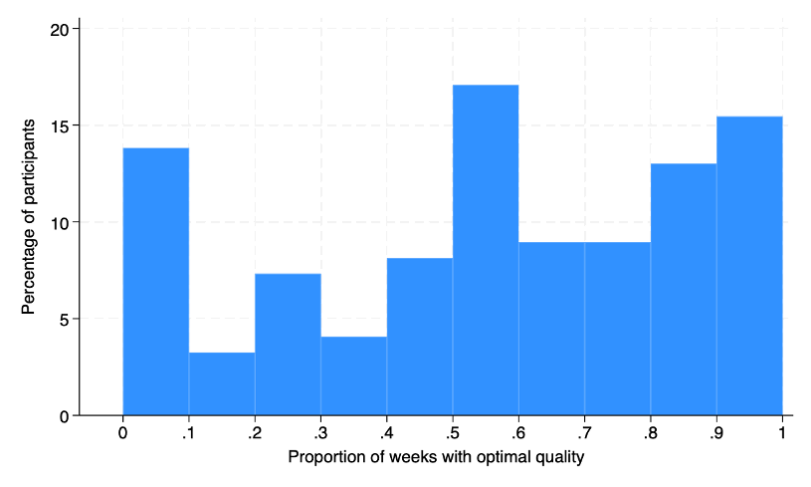
Distribution of participants by proportion of weeks with optimal data.

Overall, 31% (508/1639)of submitted data were rated as optimal quality, declining from 43.6% (65/149) in week 1 to 29.5% (44/149) in week 11. An additional 2.6% (42/1639) of submissions were rated as minimal acceptable quality, which remained relatively stable for the duration of the study. Almost exclusively, the submissions classified as poor quality seemed to be from truncated samples (see Figure 1 for an example). There was a high degree of variability across participants, as shown in Figure 4. For example, 14.1% (21/149) of participants did not have any weeks with optimal data quality, and 16.1% (24/149) of participants had optimal data quality in all 11 weeks.

### Fall Rates

During the study period, 27 (21.6%) out of 125 participants reported falling at least once and 8 (6.4%) reported more than 1 fall. Among the 27 patients who fell, 13 (48%) reported the fall through the GaitMate app in addition to follow-up, whereas the remainder were reported through phone follow-up alone. A total of 13 (48%) out of 27 patient reported a fall injury and 2 (7%) reported needing acute medical care for the fall. None of the falls occurred during the at-home TUG tests.

### Usability, Safety, and Qualitative Feedback

Among the 119 participants who completed phone follow-up, 99 (83.2%) agreed that the GaitMate app was easy to use, whereas 11 (9.2%) disagreed. A total of 114 (95.8%) agreed that they felt safe completing the at-home TUG tests, whereas only 1 (0.8%) disagreed. We identified several key barriers to completing at-home TUG tests. Several participants (n=3) related that they missed TUG test submissions because they did not have someone present with them and so could not fulfill the pretask safety checklist. A couple of participants (n=2) reported that they were busy with managing medical issues, and several (n=3) reported losing or forgetting their ID. Some participants (n=5) relayed that they either did not notice the app notification delivered each Sunday or found the “Weekly Check-in” button inactive when they had a TUG test due. Finally, some participants (n=5) stated that they accidentally tapped the “DONE” button when placing the phone in the waist belt pouch, thus prematurely ending the task for the week.

## Discussion

This study explored the feasibility of collecting weekly accelerometer data during an instrumented TUG test from older adults after ED discharge using a custom iPhone app and the factors influencing adherence to this RPM technology. Our primary finding is that, among a diverse cohort of older ED patients with fall risk, the majority of weekly at-home TUG samples were submitted, with declines over 11 weeks after discharge. We also found that about a third of submitted accelerometer data were of optimal quality. Together, these findings suggest that collecting high-quality longitudinal mobility data in older adults is challenging.

To our knowledge, this is the first report of adherence to at-home functional mobility assessments in community-dwelling older adults. The adherence found in this study is better than most prior studies using app-based remote assessments. For example, a systematic review of 99 studies examining adherence to mobile health apps, most of short duration and few including older adults, found an average adherence of 56% [15]. Comparing our findings to other RPM studies is difficult given the lack of reliable reporting on longitudinal engagement in prior work [20].

Our findings provide insights into technical and human factors that may have influenced protocol adherence. For the participants who completed no weekly tasks, there seemed to be issues around technological literacy that could not be overcome in study orientation or follow-up support phone calls. For the participants with variable adherence, factors included needing assistance to perform tasks, technical issues with the app, forgetfulness, and acute health issues during the monitoring period. The GaitMate app prioritized safety by requiring participants to have another person present during task completion which was problematic for the participants who lived alone.

Other human factors influencing adherence included lost or forgotten user IDs, missed app notifications, and distracting acute health events. To meet regulatory concerns, we required re-entry of a unique ID for each app use, but several participants reported this as an undesired barrier to completion. Our finding that notifications were often missed suggests that alternative modalities such as SMS text messaging might improve task completion rates [21]. Technical difficulties such as premature TUG test closure due to accidental screen taps could be addressed by removing that design feature or requiring verification before ending. Addressing these barriers is crucial for boosting the engagement and effectiveness of RPM interventions.

We also identified human factors affecting data quality, with the majority of poor samples likely due to inadvertent button presses prematurely terminating data logging. Future versions should consider an extra verification step before stopping data capture. Variability in quality may also indicate difficulties in properly positioning or securing the smartphone in the waist-worn pouch. These findings highlight the need to carefully balance user experience with optimal data collection in RPM. Alternative wearable technologies such as Fitbits or smartwatches could allow easier data gathering but have other limitations around compliance, charging requirements, and costs. Prior work shows that older adults have high Fitbit adherence [22], but current models lack the high-resolution accelerometry needed for detailed gait or balance analysis. Smartwatches are promising [23] but limited by the costs of these devices. Further, reliably extracting high-quality gait metrics from wrist-worn sensors is technically challenging given that arm swing is uncoupled from leg motion.

Limitations of our study include the reliance on a convenience sample from a single academic ED, which may limit the generalizability of our findings. Our sample was also younger and more male than typical ED patients who fall [24]. Additionally, the study’s design did not allow for a comparison of adherence to other types of tasks and app designs, limiting the interpretation to TUG tasks with our specific app interface. Future research should aim to address these limitations by incorporating a more diverse participant pool, extending the follow-up period, and designing real-time analytics on gait and balance after discharge to focus fall prevention efforts. Further exploration into personalized interventions and feedback mechanisms within RPM technologies and how to incorporate these into health care systems could also enhance patient engagement and adherence.

In conclusion, our study contributes to growing evidence on the potential utility of RPM in postdischarge care [25], offering insights into the practical challenges and user experiences for older adults in completing smartphone functional mobility tasks at home. Addressing the human and technological barrier we identified can enable smartphone apps and RPM to play an important role in postdischarge care to reduce fall risk in older patients.

## Supplementary material

10.2196/57601 Multimedia Appendix 1 Example of the waist belt and phone placement.

10.2196/57601 Multimedia Appendix 2 Screenshots of the GaitMate app.

## Abbreviations

ED: emergency department
RA: research associate
RPM: remote patient monitoring
STROBE: Strengthening the Reporting of Observational Studies in Epidemiology
TUG: Timed Up-and-Go
