# Supplementary figures and images for: Feasibility of Measuring Smartphone Accelerometry Data During a Weekly Instrumented Timed Up-and-Go Test After Emergency Department Discharge: Prospective Observational Cohort Study
Source: JMIR Aging. 2024 Sep 4;7:e57601. doi: 10.2196/57601 (PMC11440574; doi:10.2196/57601)

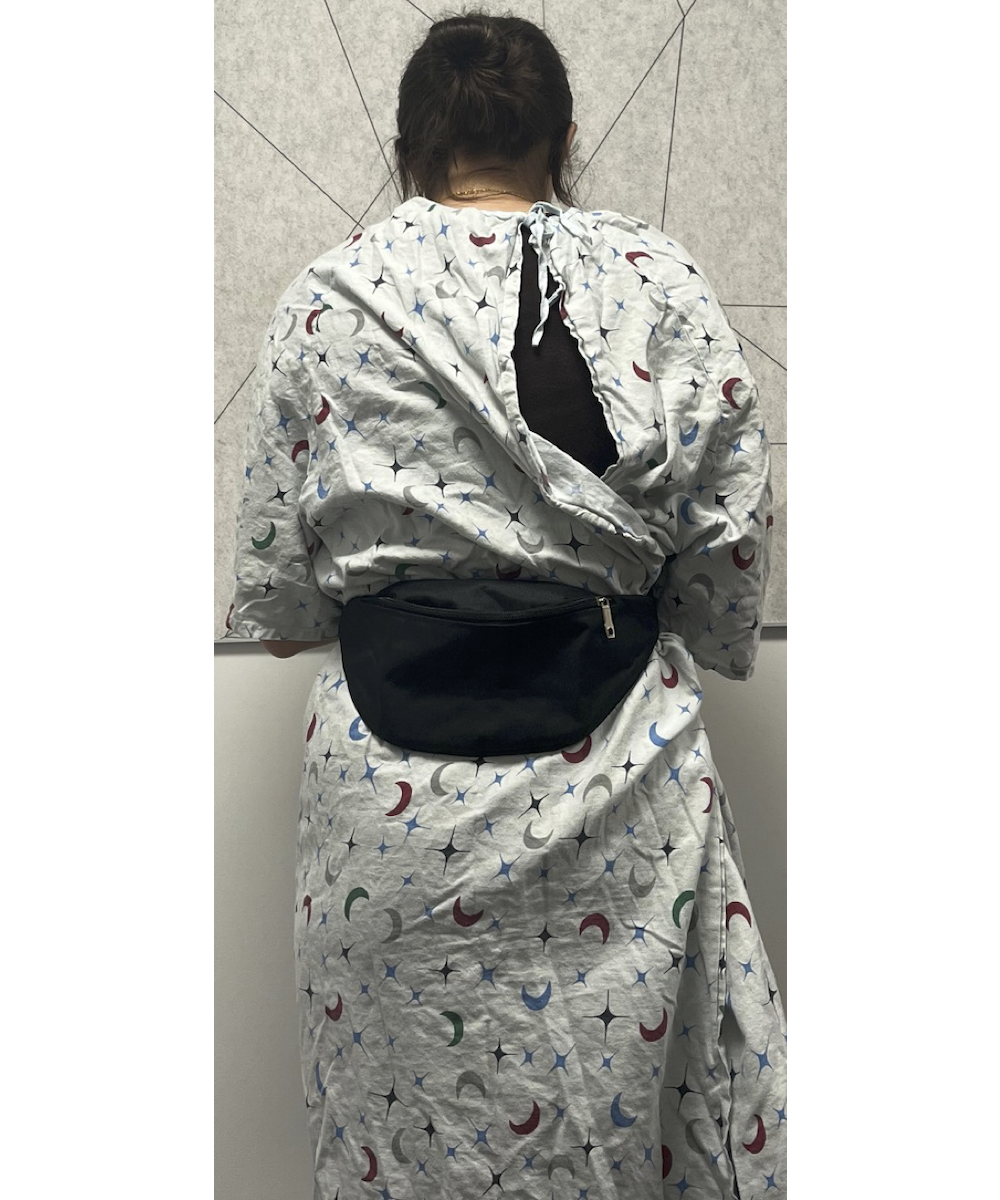

Supplement: Multimedia Appendix 1 [file aging-v7-e57601-s001.png]

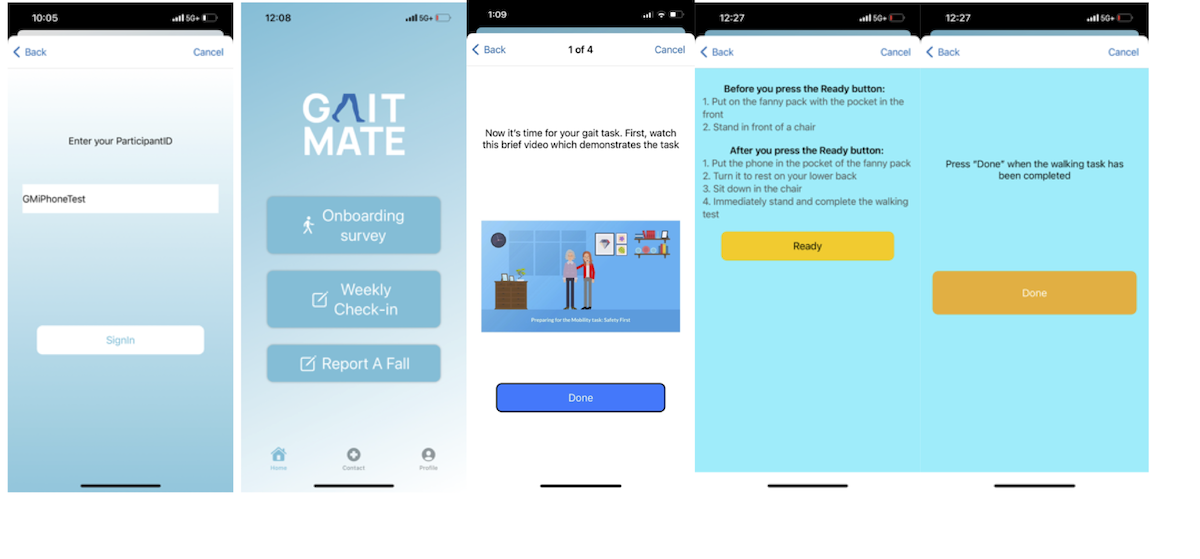

Supplement: Multimedia Appendix 2 [file aging-v7-e57601-s002.png]
